# Regulatory Mechanisms of bHLH Transcription Factors in Plant Adaptive Responses to Various Abiotic Stresses

**DOI:** 10.3389/fpls.2021.677611

**Published:** 2021-06-18

**Authors:** Yuchen Qian, Tongyao Zhang, Yan Yu, Liangpeng Gou, Jingting Yang, Jia Xu, Erxu Pi

**Affiliations:** College of Life and Environmental Sciences, Hangzhou Normal University, Hangzhou, China

**Keywords:** plant, bHLH transcription factor, flavonoids, abiotic stresses, *cis*-elements

## Abstract

Basic helix-loop-helix proteins (bHLHs) comprise one of the largest families of transcription factors in plants. They have been shown to be involved in responses to various abiotic stresses, such as drought, salinity, chilling, heavy metal toxicity, iron deficiency, and osmotic damages. By specifically binding to *cis*-elements in the promoter region of stress related genes, bHLHs can regulate their transcriptional expression, thereby regulating the plant’s adaptive responses. This review focuses on the structural characteristics of bHLHs, the regulatory mechanism of how bHLHs are involved transcriptional activation, and the mechanism of how bHLHs regulate the transcription of target genes under various stresses. Finally, as increasing research demonstrates that flavonoids are usually induced under fluctuating environments, the latest research progress and future research prospects are described on the mechanisms of how flavonoid biosynthesis is regulated by bHLHs in the regulation of the plant’s responses to abiotic stresses.

## Introduction

Basic helix-loop-helix proteins (bHLHs), are one of the largest transcription factor (TF) families. They are widely distributed in plants, fungi, and animals (Riechmann and Muyldermans, 1999; Carretero-Paulet et al., 2010). In Ludwig et al. (1989), the first plant bHLH TF was observed in maize. Since then, a great many bHLHs have been proven to regulate plant responses to various abiotic stresses. bHLHs are involved in regulating the synthesis of flavonoids (Ludwig et al., 1989), which play important roles in the ROS homeostasis under these stresses. As an illustration of the size of the bHLHs family in various plant species, 164 bHLH TFs have been found in Arabidopsis (*Arabidopsis thaliana* L.), while there are 180 in rice (*Oryza sativa* L.), 190 in tobacco (*Nicotiana tabacum* L.), 191 in grapes (*Vitis vinifera* L.), 102 in walnut (*Juglans regia* L.), 85 in *Ginkgo biloba*, 268 in *Brassica oleracea*, 440 in *Brassica napus*, and 251 *Brassica rapa* (Xiong et al., 2005; Jaillon et al., 2007; Rushton et al., 2008; Carretero-Paulet et al., 2010; Miao et al., 2020; Zhou et al., 2020; Zhao et al., 2021).

TFs, also known as *trans*-acting factors, are a category of proteins that specifically bind to *cis*-acting elements in the promoter region of eukaryotic genes. They regulate specific physiological or biochemical processes in cells at the transcriptional level. The protein structure of TFs generally contains four functional domains: a DNA-binding domain, a transcriptional regulation domain, an oligomerization site, and a nuclear localization domain. The transcriptional regulatory domain may include both an activation domain and an inhibitory domain (Yang et al., 2020).

The bHLH transcription factor conservatively contains two connected sub-regions, the N-terminal basic region directly followed by the HLH (helix-loop-helix) domain (Atchley et al., 1999). More than 50% of bHLHs that have been found in plants possess a highly conserved HER motif (His5-Glu9-Arg13) to achieve DNA binding and regulate the transcription of their target genes (Atchley and Fitch, 1997; Massari and Murre, 2000; Toledo-Ortiz et al., 2003). The HLH region is composed of 40∼50 amino acid residues, which is required for the formation of dimers (Sharker et al., 2020).

The animal bHLHs were first categorized into six subfamilies (A∼F) by Atchley et al. (1999) based on characteristics of the HLH and other conversed domains. The phylogenetic tree of plant bHLH was first constructed in *Arabidopsis thaliana*. The bHLH family of *Arabidopsis* was divided into 12 subfamilies (Gao et al., 2017). Later, these subfamilies were expanded into 32 subgroups by phylogenetic analysis based on the 638 bHLH genes extracted from *Arabidopsis*, *Populus trichocarpa*, *Oryza sativa*, *Physcomitrella patens*, and five algae species (Carretero-Paulet et al., 2010). Evolutionary and functional relationships within subfamilies are supported by intron patterns, predicted DNA-binding motifs, and the architecture of conserved protein motifs. Some subfamilies may modulate biological responses critically for the development of terrestrial plants.

## Regulatory Mechanism of bHLHs Involved in Transcriptional Activation

**FIGURE 1:**
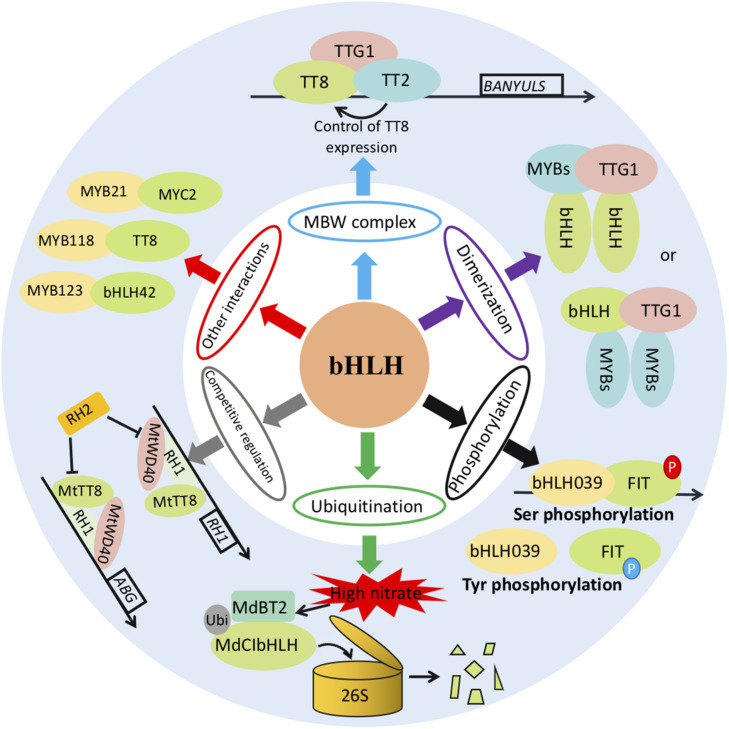
Different regulatory patterns of bHLH transcription activation.

As one of the largest TF families, bHLHs regulate the expression of downstream target genes usually in a binary or ternary complex form (Figure 1). The ternary MBW complex has been analyzed extensively in various biological processes (Dixon et al., 2005; Shoji and Hashimoto, 2011; An et al., 2012). The bHLH proteins of the IIIf subgroup (TT8, GL3, EGL3, and AtMYC1) can interact with R2R3-MYBs from various subgroups (TT2, PAP1, or PAP2), and form ternary complexes with a WD-repeat protein (TTG1) (Baudry et al., 2004). The MYB5-TT8-TTG1 complex is activated in the endothelium to regulate *DFR* (*dihydroflavonol reductase*), *LDOX* (*leucoanthocyanidin dioxygenase*), and *TT12* expression, whereas the TT2-EGL3/GL3-TTG1 complexes regulate the expression of *LDOX*, *BAN* (*BANYULS*; *anthocyanidin reductase*), *AHA10* (*autoinhibited H^+^-ATPase isoform 10*), and *DFR* in the chalaza (Xu et al., 2015). Regulatory complexes composed of MYB10, bHLH3, and WD40 may control the biosynthesis of anthocyanins in peaches (Ravaglia et al., 2013). *BoPAP2* (*MYB*), *BoTT8*, *BoEGL3.1*, *BoMYC1.2* (*bHLH*), and *BoTTG1* (*WD40*) have been identified as candidate genes for regulating anthocyanin biosynthetic activity in cabbage (Jin et al., 2018).

There are also many studies that address the activation mechanism of the MYB-bHLH binary complex (Zimmermann et al., 2004). The C-terminal domain of many bHLHs have been found to contain a MYB-binding site. Through binding to this site, MYB could heterodimerize with bHLHs (Heim et al., 2003). For example, VvMYC1 in grapes cannot activate the promoters of *CHI*, *UFGT*, and *ANR* genes in the flavonoid biosynthetic pathway by itself, but the co-transfection of *VvMYC1* with a *MYB* transcription factor can significantly activate the expression of the genes for these three enzymes (Hichri et al., 2010). *Rosea1* (*ROS1*, *MYB*-type) and *Delila* (*DEL*, *bHLH*-type) from Snapdragon (*Antirrhinum majus*) can specifically induce anthocyanin accumulation when co-expressed in tomato fruits (Outchkourov et al., 2014). Xiang et al. (2015) found that CmbHLH2 significantly upregulated *CmDFR* transcriptional expression and triggered anthocyanin accumulation when co-expressed with *CmMYB6*. Using transient assays in both *Nicotiana tabacum* leaves or *Actinidia arguta* fruits and stable transformation in *Arabidopsis*, Wang et al. (2019) demonstrated that co-expression of *AcMYB123* and *AcbHLH42* is a prerequisite for anthocyanin production by activating transcription of *AcF3GT1* and *AcANS* or their homologous genes. Li L. et al. (2019) found that AabHLH1 interacted with AaMYB3 to regulate the accumulation of procyanidine. In addition, DhMYB2 interacted with DhbHLH1 to regulate anthocyanin production in Dendrobium hybrid petals (Li et al., 2017). As well, the bHLH transcription factor PPLS1 interacts with SiMYB85 to control the color of leaf sheath and pulvinus by regulating anthocyanin biosynthesis in *Setaria italica* (Bai et al., 2020).

The transcriptional activities of bHLHs can also be regulated through dimerization, and a series of post-translational modifications including phosphorylation and ubiquitination (Figure 1; Xu et al., 2015). The *Arabidopsis* bHLH transcription factor FER-LIKE IRON DEFICIENCY-INDUCED TRANSCRIPTION FACTOR (FIT) displays a pivotal role in the Fe-deficiency response, and its activity is regulated by heterodimerizing with bHLH039 (Gratz et al., 2019). Interestingly, this heterodimerization capacity is affected by differential phosphorylation. Specifically, the heterodimerization is activated through Ser and deactivated through Tyr site phosphorylation (Gratz et al., 2020). Another *Arabidopsis* bHLH transcription factor, SPEECHLESS (SPCH), initiates the stomatal lineage (Simmons and Bergmann, 2016). Recent research revealed that the protein stability of SPCH could be positively regulated by phosphorylation mediated via the MITOGEN-ACTIVATED PROTEIN KINASE (MAPK) cascade or GSK3-like kinase BRASSINOSTEROID INSENSITIVE 2 (BIN2) protein phosphatase 2A phosphatase (Yang et al., 2015; Bian et al., 2020). *Chenopodium glaucum* CgbHLH001 can positively regulate plant stress tolerance via clearing excessive ROS and accumulating transcripts of stress-related genes (Wang et al., 2017). A recent study indicates that CgbHLH001 activity might be affected by phosphorylation through interaction with CgCDPK (Zhou et al., 2021). Ubiquitination can likewise directly regulate bHLH activity via the 26S proteasome protein degradation pathway (Figure 1). The BTB-BACK-TAZ domain protein MdBT2 in apple can target MdCIbHLH1 for ubiquitination, so as to regulate malate accumulation and vacuolar acidification (Zhang et al., 2020a, b). *Pyrus pyrifolia* CONSTITUTIVE PHOTOMORPHOGENIC1 (PpCOP1) could ubiquitinate PpbHLH64 and trigger its proteolysis, further reducing the accumulation of anthocyanins (Tao et al., 2020). bHLH activity can also be indirectly regulated by ubiquitination through the degradation of its partners in the MBW complex. The *Arabidopsis thaliana* MYB Interaction Factor 1 (AtMIF1) is a member of the ubiquitin-protein ligase E3 complex involved in the 26S proteasome protein degradation pathway. AtMIF1 ubiquitinates and degrades AtMYB5, so that transcriptional activation of the MYB/bHLH/WD-repeat (MBW) complex further increases oil content by attenuating GL2 inhibition (Cheng et al., 2021).

The latest reports reveal an emerging regulatory mechanism of MBW transcriptional activation by antagonistic interactions with MBW components (Figure 1). MYB paralogs or ROS-related proteins could disrupt the activated MBW complex by competitively interacting with bHLHs. In *Medicago truncatula*, two R2R3 MYB paralogs, RED HEART1 (RH1) and RH2, play vital roles in patterned pigmentation. These two antagonistic paralogous proteins competitively bind the MtTT8-MtWD40-1 complex to fine tune the phenotypes of leaf anthocyanin spot marking (Wang et al., 2021). In *Zea mays*, the ROS-related protein ZmSRO1e (TO RCD-ONEs) interacts with ZmPL1 (MYB)/AtPAP1 (bHLH) to inhibit the formation of an activated MBW complex, thereby repressing the over-accumulation of anthocyanins under abiotic stress (Qin et al., 2021). These two reports provide a multidimensional antagonistic regulatory paradigm for MBW activation. It’s reasonable to speculate that bHLH paralogs or other stress/growth-related proteins might also regulate the MBW activity by competitively interacting with MYB in the complex.

## bHLHs Selectively Bind to Particular *cis-*Acting Elements of Target Genes

The bHLH TFs can form homologous or heterologous dimers and bind to target genes on specific *cis*-acting elements. Among them, the most frequently studied *cis*-acting elements are the E-box (5′-CANNTG-3′) and the N-box [5′-CACG(A/C)G-3′] (Li et al., 2006). bHLHs DNA binding domains containing at least five basic amino acids in the N-terminal basic region are expected to bind these DNA motifs (Massari and Murre, 2000).

The E-box is one of the most common targets of the bHLH subfamilies 1 and 27. According to three-dimensional structural analysis of bHLHs, Glu-13 and Arg-16 are essential for E-box-binding recognition (Shimizu et al., 1997; Wang et al., 2003; Carretero-Paulet et al., 2010). This *cis*-acting element is usually located in the promoter of genes involved in multiple physiological pathways. Nims et al. (2009) found that the yew bHLH TcJAMYC can bind to the E-box in the promotor of paclitaxel-related genes to activate their transcriptional expression. Huang et al. (2013) demonstrated that the *Poncirus trifoliata* PtrbHLH can bind to an E-box in the promoter region of the *POD* gene. Wu et al. (2015) showed that the rice OsbHLH062 can bind to an E-box in the promoter of ion transport genes such as *OsHAK21*. Zhang et al. (2017) found that PnbHLH1 can interact with the E-box core sequence in *Panax notoginseng*, a traditional Chinese medicine, to induce the triterpenoid saponins. Geng and Liu (2018) experimentally showed that CsbHLH18 bound to the promoter of the sweet orange (*Citrus sinensis*) *CsPOD* gene through the E-box. Recent studies have found that GhbHLH18 strongly binds to the E-box in the promoter region of the *GhPER8* gene coding for a peroxidase from cotton (Gao et al., 2019). Finally, Chakraborty et al. (2019) demonstrated through DNA-protein interactions both *in vitro* and *in vivo* that the bHLH transcription factor MYC2 can bind to the *cis*-acting E-box element in the HY5 promoter to negatively regulate *HY5* expression.

A specific member of the E-box family, the G-box (5′-CA**CG**TG-3′) could be recognized by about 81% of bHLHs (Toledo-Ortiz et al., 2003; Li et al., 2006; Pires and Dolan, 2010) mainly from subfamilies 4, 5, 10, 11, 13, 14, 24, 25, and 26 (Bovy et al., 2002; Yi et al., 2005; Carretero-Paulet et al., 2010; Fu et al., 2014; Chen et al., 2018; Gao et al., 2018; Jiang et al., 2018). Qian et al. (2007) confirmed the specific interaction between the pea bHLH transcription factor PsGBF and the G-box of *PsCHS1* by transient expression experiments in tobacco. In *Arabidopsis*, the bHLH transcription factor PIL5 has a high affinity with the G-box as well (Kang et al., 2010). *Arabidopsis* bHLH106 was characterized through its association with salt tolerance. Transcriptional analysis of an *AtbHLH106* overexpression line showed 198 genes positively were regulated and 36 genes were negatively regulated; these genes possessed one or more G-box sequences in their promoter regions, and many of them are associated with the abiotic stress response (Ahmad et al., 2015). Zhang et al. (2011) isolated *CrMYC2* from a *Catharanthus roseus* cDNA library and found that its corresponding protein could specifically bind to G-box of the *ORCA3* gene. In addition, tobacco NtMYC2 and NtbHLH123 bind directly to the G-box region of the *PMT2* and *CBF*, respectively, and activated their transcription (Shoji and Hashimoto, 2011; Zhao et al., 2018).Besides, *Tamarix hispida* ThbHLH1 specifically binds to the G-box of *P5CS* and the *ALDH* gene (Ji et al., 2016). The apple MdMYC2 homodimer also binds to the G-box motif of the *AtJAZ3* gene (An et al., 2016). Moreover, the *Hevea brasiliensis* bHLHs HbMYC2, HbMYC3, and HbMYC4 of interacted with the G-box of the *HbPIP2* promotor (Zhai et al., 2018).

The N-box is associated with bHLHs possessing an enhancer activity. bHLHs that bind to the N-box usually have a proline in the N-terminal basic domain and a “WRPW” sequence at its C-terminus (Cordeiro et al., 2016). OsPIF14 binds to the *OsDREB1B* promoter on two N-boxes [CACG(A/C)G] (Cordeiro et al., 2016). Diterpenoid phytoalexin factor (DPF) is a rice bHLH transcription factor which positively regulates *CPSCPS2* transcription through N-box binding. DPF can also regulate *CYP99A2* through the N-box, thereby affecting the biosynthesis of diterpenoid phytoalexins (DP) (Yamamura et al., 2015). In addition, some bHLHs can bind to other *cis*-acting elements, for example, AtbHLH112 can not only bind the E-box, but also bind the GCG-box [5′-GG(G/T)CC(G/T)(GA)(TA)C-3′] (Liu et al., 2015).

Heterodimers can be formed between different bHLHs. Fairchild et al. (2000) discovered that two bHLH TFs, PIF3/AtbHLH008 and HFR1, could heterodimerize. This interaction effectively prevented monomer PIF3/AtbHLH008 from binding to the E-box of *phytochrome A* (*phyA*), thereby modulating phyA signaling. In addition, bHLHs can heterodimerize with other TFs, such as R2R3-MYB/BZR1-BES1 as well as other signal transduction proteins (Yin et al., 2005; Dubos et al., 2008). The formation of heterodimers makes it possible for different TFs to interact with each other to co-regulate the expression of target genes.

In summary, bHLHs bind directly to *cis*-elements in the promoter of target genes or form heterodimers to co-regulate their expression. To date, E-box *cis*-acting elements, in particular, the G-box of this family, are the widest binding targets of bHLHs (Table 1). We found that most bHLHs in subfamilies 1, 2, 12, 13, 15, 19, 24, 25, 26, 27 can bind to the G-box; and members in subfamilies 1, 2, 13, 15, 22, 26, 27 can recognize other *cis*-elements in the E-box family, except for the G-box; members of subgroup 15 could also bind to the GCG-box (Abe et al., 1997; Wang et al., 2013; Gao et al., 2016; Yao et al., 2018; Xi et al., 2021).

**TABLE 1:** Mechanism of bHLH on transcriptional regulation of target genes.

*cis*-acting element	Plant species	Nomenclature	Subfamily	Effect	Referenes
E-box, G-box	*Arabidopsis thaliana* L.	AtbHLH122	27	Participate in drought resistance, salt resistance, osmotic resistance	Liu et al., 2014
E-box, GCG-box	*Arabidopsis thaliana* L.	AtbHLH112	15	Participate in drought resistance, salt resistance	Liu et al., 2015
G-box	*Arabidopsis thaliana* L.	AtbHLH106	13	Participate in salt resistance	Ahmad et al., 2015
G-box	*Arabidopsis thaliana* L.	PIL5/AtbHLH15	24	Unknown	Kang et al., 2010
E-box, G-box	*Arabidopsis thaliana* L.	AtMYC2/AtbHLH006	2	Involved in niche regulation of root stem cells	Chen et al., 2011; Qi et al., 2015
E-box, G-box	*Oryza sativa* L.	OsbHLH096	26	Increase phosphorus hunger tolerance	Yi et al., 2005
N-box	*Oryza sativa* L.	DPF/OsbHLH025	7	Affect DP biosynthesis	Yamamura et al., 2015
E-box	*Poncirus trifoliata*	PtrbHLH	1	Scavenging oxygen free radicals, participate in low temperature resistance	Huang et al., 2013
E-box	*Gossypium hirsutum* Linn.	GhMYC4	2	Participate in drought resistance, salt resistance	Gao et al., 2016
E-box	*Gossypium hirsutum* Linn.	GhbHLH18	22	Regulate the content of coniferyl alcohol and sinapic alcohol	Gao et al., 2019
G-box	*Catharanthus roseus* (L.) G. Don	CrMYC2	2	Participates in the biosynthesis of terpenoid indole alkaloids	Zhang et al., 2011
G-box	*Nicotiana tabacum* L.	NtMYC2	2	Regulate nicotine biosynthesis	Shoji and Hashimoto, 2011
E-box, G-box	*Nicotiana tabacum* L.	NtbHLH123	15	Participate in cold resistance	Zhao et al., 2018
G-box	*Betula platyphylla* Suk.	BpbHLH7	12	Participates in the synthesis of triterpenoids	Wang et al., 2018
		BpbHLH8	25		
E-box	*Citrus sinensis* (L.) Osbeck	CsbHLH18	13	Scavenging oxygen free radicals, participate in low temperature resistance	Geng and Liu, 2018
E-box, G-box	*Malus* × *domestica* Borkh.	MdCIbHLH1	Orphan	Participate in flower bud dormancy and dormancy removal	Ren et al., 2016
G-box	*Malus* × *domestica* Borkh.	MdMYC2	2	Participate in the Jasmonic acid (JA) signaling pathway	An et al., 2016
G-box	*Medicago truncatula*	MtbHLH148	19	Adjust the optical signal response	Wang et al., 2019
G-box	*Tamarix hispida* Willd.	ThbHLH1	26	Reduce the accumulation of reactive oxygen species (ROS)	Ji et al., 2016
G-box	*Pisum sativum* Linn.	PsGBF	1	Unknown	Qian et al., 2007

## bHLHs Regulate Plant Responses to Various Abiotic Stresses

Previous studies found that bHLHs were involved in a variety of pathways regulating the adaptation to stress in plants, including resistance to mechanical damage, drought, high salt, oxidative stress, low temperature stresses, heavy metal stress, iron deficiency, and osmotic stress (Babitha et al., 2015; Zhao et al., 2016). One of the most common responses of plants to stress is to enhance the biosynthesis of different types of compatible organic solutes. In general, this mechanism protects plants from stresses in multiple different ways through regulating cell osmotic homeostasis, eliminating excessive ROS, maintaining the integrity of the plasma membrane, and stabilizing enzymes and structural proteins (Esmaeilpour et al., 2015).

**FIGURE 2:**
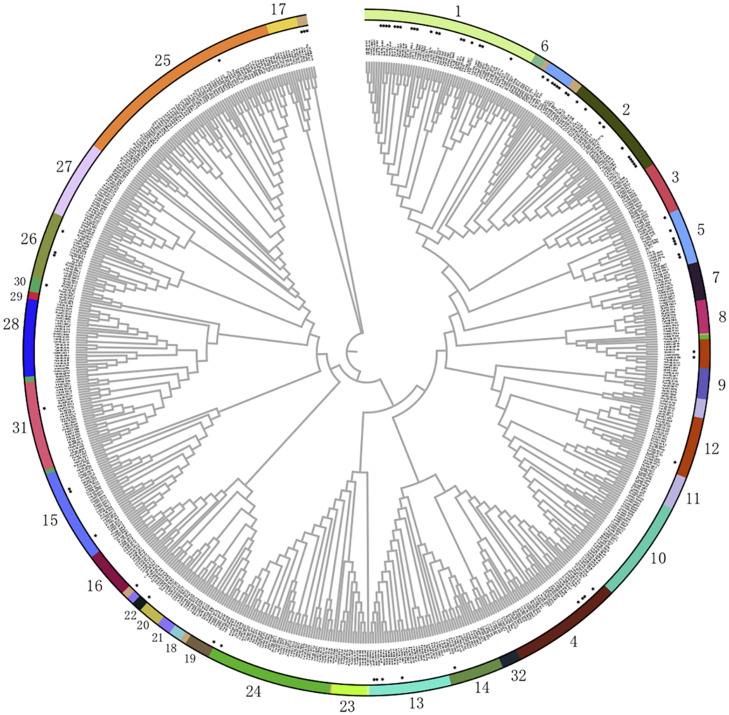
Phylogenetic relationships of bHLH family members. The phylogenetic tree was constructed using MEGA7.0 with the neighbor-joining method and 1000 bootstrap replicates (Huang and Dai, 2015; Wang et al., 2016b). Then the trees were visualized using iTOL (https://itol.embl.de/). Group names were marked outside the circle. The bHLH protein sequences were downloaded from the JGI (http://www.jgi.doe.gov/) and NCBI (https://www.ncbi.nlm.nih.gov) databases. The Gene and Protein IDs of all these bHLHs are list in Supplementary Tables 1, 2. And the bHLH members that have been functional annotated are labeled with a star marker.

To uncover the potential roles of bHLHs from different subfamilies in various stress responses, most of the functionally annotated bHLH TFs were categorized into the abovementioned 32 subfamilies (Figure 2 and Table 2). The response function of different bHLH subfamilies to abiotic stress is different. We found that nearly half of the bHLHs subfamilies participated in abiotic stress responses. Among them, there are nine subfamilies of bHLHs involved in drought response, including subfamilies 1, 2, 4, 5, 13, 15, 24, 26, and 30, in which most members are from subfamilies 1, 2, and 15. As well, there are nine subfamilies of bHLHs participating in salinity resistance, including subfamilies 1, 2, 4, 5, 7, 13, 15, 26, and 27. The majority of salinity responsive bHLHs are from subfamilies 1 and 4. In addition, five subfamilies were found to regulate the cold tolerance of plants, including 1, 2, 13, 15, and 26. Interestingly, members in subfamily 1 were found to be able to take over the regulation of chilling responses. Furthermore, bHLHs of subfamilies 1, 3, 4, 12, 28, and 31 are involved in the response to iron deficiency. In short, bHLHs of subfamily 1 seem to participate in most kinds of abiotic stresses, such as drought, salinity, chilling and iron deficiency. Moreover, members of subfamily 2 mainly participate in drought, salt, and cold stress, while that of subfamily 4 mainly regulates responses to drought, salt, and iron deficiency stress.

**TABLE 2:** bHLH transcription factors involved in plant abiotic stress response.

Original plant	Stress response	Nomenclature	Subfamily	Target gene	Regulation type	Function	References
*Arabidopsis thaliana* L.	Drought	rd22BP1/AtMYC2/AtbHLH006	2	*rd22/P5CS1/AtNHX1*	Positive regulation	Participate in drought stress response	Abe et al., 1997; Chen et al., 2011; Verma et al., 2020
*Arabidopsis thaliana* L.	Drought	AtbHLH112	15	*P5CS*	Positive regulation	Participate in drought stress response	Liu et al., 2014, 2015
*Arabidopsis thaliana* L.	Drought	AtbHLH68	15	Unknown	Unknown	Participate in drought stress response	Le Hir et al., 2017
*Arabidopsis thaliana* L.	Drought/Salt	AtAIB/AtbHLH17	2	Unknown	Positive regulation	Participate in drought and salt stress response	Liu et al., 2014, 2015
*Arabidopsis thaliana* L.	Salt	AtNIG1/AtbHLH028	2	Unknown	Positive regulation	Participate in salt stress response	Kim and Kim, 2006
*Arabidopsis thaliana* L.	Salt	AtbHLH92	7	Unknown	Unknown	Participate in salt stress response	Jiang et al., 2009
*Arabidopsis thaliana* L.	Salt	AtbHLH122	27	*AtNHX6*	Unknown	Participate in salt stress response	Liu et al., 2014; Krishnamurthy et al., 2019
*Arabidopsis thaliana* L.	Cold	AtICE1/AtbHLH116	1	*CBF*	Positive regulation	Participate in cold stress response	Chinnusamy et al., 2003
*Arabidopsis thaliana* L.	Iron deficiency	FIT/AtbHLH29	1	Unknown	Unknown	Participate in iron deficiency response	Wang et al., 2007, 2013; Yuan et al., 2008; Lingam et al., 2011; Cui et al., 2018
		AtbHLH38	12	*FRO2, IRT1*	Positive regulation		
		AtbHLH39	12	*FRO2, IRT1*	Positive regulation		
		AtbHLH100	12	Unknown	Positive regulation		
		AtbHLH101	12	Unknown	Positive regulation		
*Arabidopsis thaliana* L.	Iron deficiency	AtbHLH18	3	Unknown	Negative regulation	Participate in iron deficiency response	Cui et al., 2018
		AtbHLH19	3	Unknown	Negative regulation		
		AtbHLH20	3	Unknown	Negative regulation		
		AtbHLH25	3	Unknown	Negative regulation		
*Arabidopsis thaliana* L.	Iron deficiency	AtbHLH104	4	Unknown	Positive regulation	Participate in iron deficiency response	Li X. et al., 2016; Rohrmann, 2019
*Arabidopsis thaliana* L.	Iron deficiency	AtbHLH34	4	Unknown	Unknown	Participate in iron deficiency response	Li X. et al., 2016
*Arabidopsis thaliana* L.	Iron deficiency	AtbHLH121	4	Unknown	Unknown	Participate in iron deficiency response	Lei et al., 2020
*Arabidopsis thaliana* L.	Iron deficiency	ILR3/AtbHLH105	4	Unknown	Positive regulation/Negative regulation	Participate in iron deficiency response	Long et al., 2010; Kroh and Pilon, 2019; Tissot et al., 2019
		PYE/AtbHLH47	4	Unknown	Unknown	Participate in iron deficiency response	
*Arabidopsis thaliana* L.	Iron deficiency	AtbHLH115	4	Unknown	Unknown	Participate in iron deficiency response	Liang et al., 2017
*Oryza sativa* L.	Salt	OsbHLH035	13	Unknown	Unknown	Participate in salt stress response	Chen et al., 2018
*Oryza sativa* L.	Salt	OsbHLH062	4	*OsHAK21*	Unknown	Participate in salt stress response	Wu et al., 2015
*Oryza sativa* L.	Salt	OsbHLH068	15	Unknown	Positive regulation	Participate in salt stress response	Huang et al., 2015
*Oryza sativa* L.	Cold	OsbHLH1	1	Unknown	Positive regulation	Participate in cold stress response	Wang et al., 2003
*Oryza sativa* L.	Iron deficiency	OsIRO2/OsbHLH056	12	OsNAS1, OsNAS3, OsIRT1, OsFDH, OsAPT1, IDS3	Positive regulation	Participate in iron deficiency response	Ogo et al., 2006
*Oryza sativa* L.	Iron deficiency	OsbHLH133	28	Unknown	Negative regulation	Participate in iron deficiency response	Kool et al., 1994
*Oryza rufipogon*	Salt/Cold	OrbHLH001	1	Unknown	Positive regulation	Participate in salt and cold stress response	Li et al., 2010
*Oryza rufipogon*	Salt/Osmotic	OrbHLH2	1	*DREB1A/CBF3, RD29A, COR15A, KIN1*	Positive regulation	Participate in salt stress and osmotic stress response	Zhou et al., 2009
*Populus euphratica*	Drought	PebHLH35	1	Unknown	Positive regulation	Participate in drought stress response	Dong et al., 2014
*Eleusine coracana* L.	Drought/Salt/Oxidative	EcbHLH57	4	Unknown	Unknown	Participate in drought, salt stress and oxidative response	Babitha et al., 2015
*Zea mays L.*	Drought	ZmPTF1	26	*NCED*, *CBF4*, *ATAF2/NAC081*, *NAC30*	Positive regulation	Participate in drought stress response	Li Z.X. et al., 2019
*Zea mays L.*	Drought	ZmPTF3	24	Unknown	Positive regulation	Participate in drought stress response	Gao et al., 2018
*Zea mays L.*	Heavy metal	ZmbHLH105	4	Unknown	Positive regulation	Participate in heavy metal stress response	Sun et al., 2019
*Vitis vinifera*	Drought/Salt/Cold	VvbHLH1	1	CBF3, RD29A	Positive regulation	Participate in drought, salt stress and cold response	Wang et al., 2016a
*Fagopyrum tataricum*	Drought/Oxidative	FtbHLH3	1	Unknown	Unknown	Participate in drought stress and oxidative response	Yao et al., 2017
*Fagopyrum tataricum*	Cold	FtbHLH2	2	Unknown	Positive regulation	Participate in cold stress response	Yao et al., 2018
*Antirrhinum majus* L.	Drought/Salt	AmDEL	5	Unknown	Positive regulation	Participate in drought and salt stress response	Wang et al., 2016b
*Solanum tuberosum*	Drought	StbHLH45	13	Unknown	Unknown	Participate in drought stress response	Wang et al., 2018
*Triticum aestivum* L.	Cold	TabHLH1	26	PT, NRT, AEs	Positive regulation	Participate in cold stress and osmotic stress response	Yang et al., 2016
*Triticum aestivum* L.	Osmotic	TabHLH39	orphan	Unknown	Unknown	Participate in osmotic stress response	Zhai et al., 2016
*Triticum aestivum* L.	Salt	TabHLH13	4	Unknown	Unknown	Participate in salt stress response	Fu et al., 2014
*Solanum lycopersicum*	Salt/Osmotic	SlICE1a	1	Unknown	Positive regulation	Participate in salt stress and osmotic stress response	Feng et al., 2013
*Solanum lycopersicum*	Iron deficiency	FER	1	Unknown	Unknown	Participate in iron deficiency response	Ling et al., 2002
*Tamarix hispida* Willd.	Salt	ThbHLH1	26	Unknown	Positive regulation	Participate in salt stress response	Ji et al., 2016
*Pyrus ussuriensis*	Cold	PuICE1	1	*PuDREBa*	Unknown	Participate in cold stress response	Huang et al., 2015
*Pyrus ussuriensis*	Cold	PubHLH1	1	Unknown	Positive regulation	Participate in cold stress response	Jin et al., 2016
*Raphanus sativus*	Cold	RsICE1	1	Unknown	Positive regulation	Participate in cold stress response	Man et al., 2017
*Brassica campestris L.*	Cold	BcICE1	1	Unknown	Positive regulation	Participate in cold stress response	Zhang et al., 2018
*Dimocarpus longan* Lour.	Cold	DlICE1	1	Unknown	Positive regulation	Participate in cold stress response	Yang et al., 2019
*Nicotiana tabacum L.*	Cold	NtbHLH123	15	*NtCBF*	Positive regulation	Participate in cold stress response	Zhao et al., 2018
*Citrus sinensis*	Cold	CsbHLH18	13	*CsPOD*	Positive regulation	Participate in cold stress response	Geng and Liu, 2018
*Zoysia japonica*	Cold	ZjICE1	1	*ZjDREB1*	Positive regulation	Participate in cold stress response	Zuo et al., 2019
*Vitis amurensis*	Cold	VabHLH1	1	*CBF3, RD29A*	Positive regulation	Participate in cold stress response	Xu et al., 2014
*Glycine Max* (L.) Merrill	Iron deficiency	GmbHLH57	1	Unknown	Unknown	Participate in iron deficiency response	Li L. et al., 2018
*Glycine Max* (L.) Merrill	Iron deficiency	GmbHLH300	12	Unknown	Unknown	Participate in iron deficiency response	Li L. et al., 2018
*Malus* × *domestica* Borkh.	Iron deficiency	MdbHLH104	31	*MdAHA8*	Positive regulation	Participate in iron deficiency response	Zhao et al., 2016
*Populus tomentosa* Carr.	Iron deficiency	PtFIT	1	Unknown	Positive regulation	Participate in iron deficiency response	Huang et al., 2015
*Chrysanthemum morifolium*	Iron deficiency	CmbHLH1	4	Unknown	Unknown	Participate in iron deficiency response	Zhao et al., 2014
*Hordeum vulgare subsp*. vulgare	Iron deficiency	HvIRO2	12	Unknown	Unknown	Participate in iron deficiency response	Ogo et al., 2006

### bHLHs Involved in Drought Stress

The effects of drought stress on plants are mainly manifested in decreased photosynthesis efficiency, disordered hormone metabolism, and reduced enzyme activities, causing irreversible damage to plant growth and yield (Sun et al., 2018). The bHLH TFs regulate the plant drought-tolerant responses mainly through modulation of the sensitivity to Abscisic acid (ABA) or by regulating the development of stomata, leaf trichome, and root hair (Castilhos et al., 2014). Li et al. (2007) found that the expression of the bHLH-type transcription factor *AtAIB* was temporarily induced by ABA, and that plants overexpressing *AtAIB* showed a stronger drought tolerance. Le Hir et al. (2017) found that AtbHLH68 may play a role in responding to drought stress, likely through an ABA-dependent pathway by either directly or indirectly regulating components of ABA signaling and/or metabolism. Yao et al. (2017) demonstrated that heterologous expression of tartary buckwheat *FtbHLH3* in Arabidopsis could positively regulate drought/oxidative stress tolerance in an ABA dependent manner. Rao et al. (2020) reported that a large number of bHLHs are involved in ABA signaling and positively regulated stress resistance in *Lycium ruthenicum*.

In *Arabidopsis*, AtbHLH006/rd22BP1/AtMYC2 can regulate the expression of *RD22*. Interestingly, AtbHLH006 can bind to different *cis*-acting elements under different conditions. AtbHLH006 binds to the E-box (but not G-box) on the *PLETHORA* gene promoter in JA mediated regulation of the *Arabidopsis* root stem cell niche (Chen et al., 2011); it could also bind to the G-box motif of the *SAG29* promoter to activate its expression during JA mediated leaf senescence (Qi et al., 2015). Another bHLH transcription factor, ZmPTF1 in maize, regulates drought tolerance by promoting root development and the synthesis of ABA (Li Z.X. et al., 2019). Dong et al. (2014) found that plants overexpressing *Populus euphratica PebHLH35* had significantly lower stomatal density and decreased stomatal opening than wild-type plants, so their transpiration was significantly reduced. Zhao et al. (2020) illustrated that Apple MdbHLH130 acts as a positive regulator of water stress response by regulating stomatal closure and ROS-scavenging in tobacco.

### bHLHs Involved in Salt Stress

High salt stress caused by ionic and osmotic stressors eventually results in the suppression of plant growth and a reduction in crop productivity. Some bHLH TFs have a regulatory effect under saline conditions. Under salt stress, plant cells successively face the challenges of osmotic stress, ion toxicity, and oxidative stress (Rozema and Flowers, 2008). As discussed previously, bHLH TFs usually enhance a plant’s resistances to these secondary stresses. AtNIG1 in *Arabidopsis* was the first bHLH transcription factor shown to be involved salt stress signaling pathways from plants. The survival rate and dry weight of the *atnig1* mutant decreased significantly under salt stress (Kim and Kim, 2006). Fu et al. (2014) showed that the expression level of the wheat (*Triticum aestivum* L.) *TabHLH13* gene was significantly up-regulated with the increase of salt ion concentration in the environment. Wu et al. (2015) showed that OsbHLH062 could regulate ion transport genes, such as *OsHAK21*, and modulate the Jasmonic acid (JA) signaling pathway, so as to endow plants with some resistance to salt stress. One of the most important mechanisms by which bHLH TFs participate in salt tolerance is to regulate ROS balance by directly regulating the expression of a group of peroxidase genes. Jiang et al. (2009) found that AtbHLH92 elevated *Arabidopsis* tolerance to salt and osmotic stresses through a partial dependence on ABA and SOS2. The beet homolog of *AtbHLH92*, *BvbHLH92*, was found to be expressed in both the beet root and leaf in response to salt stress (Jiang et al., 2009). In *Tamarix hispida*, *ThbHLH1* was highly expressed under high salt induction, significantly increasing peroxidase (POD) and superoxide dismutase (SOD) activities (Ji et al., 2016). AtbHLH112 increased the expression of the *POD* and *SOD* genes, while concomitantly reduced the expression of the *P5CDH* and *ProDH* genes, so as to enhance the resistance of *Arabidopsis* to high salt (Liu et al., 2014). In addition, bHLHs could also regulate the accumulations of resistance-related secondary metabolites in order to improve plant salinity tolerance. Verma et al. (2020) found that salt stress activated AtMYC2 through a mitogen-activated protein kinase (MAPK) cascade. Then, the AtMYC2 could bind to the promotor of rate-limiting enzyme *P5CS1* in the biosynthesis of proline, thereby regulating the biosynthesis of proline, and thus regulating salt tolerance. AtbHLH122 inhibited the expression of *CYP707A3* gene under NaCl stress (Liu et al., 2014). Furthermore, Krishnamurthy et al. (2019) identified AtMYC2 and AtbHLH122 as upstream regulators of the ABA-mediated AtNHX1 and AtNHX6, which are both Na^+^/H^+^ exchangers, by chromatin immunoprecipitation. Moreover, overexpression of *EcbHLH57* can enhance the resistance of tobacco to salt stress by elevating the expression of stress responsive genes such as *LEA14*, *rd29A*, *rd29B*, *SOD*, *APX*, *ADH1*, *HSP70*, and also *PP2C* (Babitha et al., 2015). OsbHLH068 has a similar effect as AtbHLH112 in regulating the salt stress response. In *Arabidopsis*, heterogenic overexpression of *OsbHLH068* can reduce salt-induced accumulation of H_2_O_2_ (Chen et al., 2017).

### bHLHs Adjust Plants to Low Temperature Stress

Cold stress is a severe threat for plant productivity and crop production, particularly so when it occurs during the growth phase (Albertos et al., 2019). The *DREB1/CBF* (*dehydration-responsive element binding/C-repeat binding factors*) gene is considered as the main regulator of plant’s cold stress response. Under low temperature stress, *OsDREBL* and *OsTPP1* genes were significantly up-regulated at the transcriptional level after overexpression of *RsICE1* (*inducer of CBF expression*) in rice, indicating that RsICE1 is involved in the DREB1/CBF cold regulation signaling network (Man et al., 2017). In *Arabidopsis* the AtICE1/AtbHLH116 protein binds to the *CBF* promoter region at low temperatures to affect transcription initiation, and *AtICE1*/*AtbHLH116* overexpressing plants showed higher tolerance to cold (Chinnusamy et al., 2003). The bHLH transcription factor DlICE1 from *Dimocarpus longan* Lour has a positive regulatory effect on cold tolerance. Overexpression of *DlICE1* in *Arabidopsis* conferred enhanced cold tolerance via increased proline content, decreased ion leakage, and reduced malondialdehyde (MDA) and reactive oxygen species (ROS) accumulation. The expressions of the ICE1-CBF cold signaling pathway genes, including *AtCBF1/2/3* and cold-responsive genes (*AtRD29A*, *AtCOR15A*, *AtCOR47*, and *AtKIN1*), were also significantly higher in *DlICE1*-overexpressing lines than in wild-type (WT) plants under cold stress (Yang et al., 2019). ICE1 proteins in other plants have similar functions. PuICE1 of can elevate the transcriptional expression of *PuDREBa* by interacting with PuHHP1, thus improving the cold resistance of *Pyrus ussuriensis* (Huang et al., 2015). The overexpression of *BcICE1* in tobacco can positively regulate the expression of stress-related genes such as *CBFs* (C-repeat binding factor) and enhance the antioxidant activity and osmotic ability of plants (Zhang et al., 2018). Zuo et al. (2019) showed that the transgenic *Arabidopsis* with overexpressed *Zoysia japonica ZjICE1* showed an enhanced tolerance to cold stress with an increase in SOD, POD, as well as higher free proline content and decreased MDA content. They also upregulated the transcript abundance of cold-responsive genes (*CBF1*, *CBF2*, *CBF3*, *COR47A*, *KIN1*, and *RD29A*). ZjICE2 from *Zoysia japonica* enhanced the tolerance of transgenic plants to cold stress by activating DREB/CBF regulators and enhancing reactive oxygen species scavenging (Zuo et al., 2019). In addition to ICE proteins, some bHLH proteins are also responsible for plant resistance to low temperatures. As a homolog of *ICE1*, the rice *OrbHLH001* could enhance the tolerance of transgenic *Arabidopsis* to freezing stress. However, the function of OrbHLH001 was different from that of ICE1 and is independent of a CBF/DREB1 cold-response pathway (Li et al., 2010). The grape *VvbHLH1* and *VabHLH1* are positive regulators of the cold stress response, and the overexpression of these two genes could enhance the expression level of the *COR* gene (Xu et al., 2014). The apple (*Malus domestica* Borkh.) *MdCIbHLH1* gene was recently identified as a hub in the transcriptional regulation of bark freezing tolerance Liang et al. (2020). Moreover, Zhao et al. (2018) demonstrated that NtbHLH123 is a transcriptional activator that plays a positive regulatory role in cold tolerance by activating the reactive oxygen species scavenging-related gene *NtCBF*. Geng and Liu (2018) found that CsbHLH18 can regulate ROS homeostasis at least partially by directly regulating the antioxidant gene *CsPOD*, thus playing an active role in cold tolerance.

### bHLHs Enhances Plant Survival in Heavy Metal Toxicity

Excessive heavy metals in agricultural lands cause declines in crop productivity (Thao et al., 2015). Song et al. (2014) found that excessive expression of *GmbHLH30* in tobacco can enhance its resistance to aluminum toxicity through the maintenance of osmotic pressure. Manganese (Mn) toxicity is also an important factor for limiting crop production in acidic soils. Sun et al. (2019) found that ZmbHLH105 may improve maize tolerance to Mn stress by regulating antioxidant mechanism-mediated ROS clearance and the expression of Mn/Fe-related transporters in plants.

### bHLHs Help Withstand Plant Iron and Copper Homeostasis

The key bHLH transcription factor in iron (Fe) uptake, FER-LIKE IRON DEFICIENCY-INDUCED TRANSCRIPTION FACTOR (FIT), is critical for adjusting Fe acquisition to plant growth and environmental constraints (Gratz et al., 2020). Kool et al. (1994) found that the bHLH133 transcription factor in rice can regulate the transport of Fe from roots to young leaves, revealing the important role of bHLH proteins in maintaining iron homeostasis in plant cells. Ling et al. (2002) found that the bHLH transcription factor FER from tomato played a role in the regulation of plant root iron nutrition. Yuan et al. (2008) found that the *FIT/bHLH29* (FER-like iron deficiency-induced transcription factor) gene played an important role in maintaining intracellular Fe balance through regulating the expression of downstream iron absorption genes. The plant hormone ethylene is one of the signals that trigger iron deficiency responses at both the transcriptional level and the post-transcriptional level. Through ethylene signal transduction, the FIT/bHLH29 protein is protected from degradation by proteasomes (Lingam et al., 2011). Increased FIT levels subsequently leads to the high level of expression of genes required for Fe acquisition. Cui et al. (2018) found that the bHLH18, bHLH19, bHLH20, and bHLH25 in the Iva subgroup are FIT/bHLH29 interactors, which can promote JA induced FIT/bHLH29 protein degradation. Previously, Wang et al. (2017, 2019) demonstrated that four Ib bHLH genes (*AtbHLH38*, *AtbHLH39*, *AtbHLH100*, and *AtbHLH101*) played important roles in the iron-deficiency responses, though they are not induced by FIT/bHLH29 under iron deficiency conditions. Actually, these four Iva bHLHs mainly antagonized Ib bHLHs, so as to regulate the stability of the FIT/bHLH29 protein under iron deficiency conditions. Furthermore, Lei et al. (2020) found that bHLH121 interplayed with another bHLH transcription factor in the Ivc subgroup to positively regulate *FIT/bHLH29* expression, thus playing a key role in maintaining Fe homeostasis in *Arabidopsis*. Zhang et al. (2015) identified the *Arabidopsis* AtbHLH104 as a member in subfamily Ivc and demonstrated that bHLH104 acted as a key component positively regulating Fe deficiency responses via targeting Ib subgroup bHLH genes and *PYE/bHLH47* expression. Li X. et al. (2016) supplemented this study and proposed that bHLH34, bHLH104, and bHLH105 (IAA-LEUCINE RESISTANT3) can be used as homologous dimers or heterodimers to regulate the stable state of Fe without redundancy. ILR3/bHLH105, alone was shown to stimulate Fe uptake by inhibiting ferritin expression (Kroh and Pilon, 2019; Tissot et al., 2019). Long et al. (2010) showed through chromatin immunoprecipitation-on-chip analysis and transcriptional profiling that PYE/bHLH47 helped maintain iron homeostasis by regulating the expression of known iron homeostasis genes. In addition, AtbHLH115 is also a positive regulator of iron deficiency responses, being negatively regulated by the E3 ligase BTS (Liang et al., 2017). In poplar, transgenic line (TL2) overexpressing *PtFIT* showed a higher chlorophyll content and Chl a/b ratio than the control plant under the conditions of iron deficiency, indicating that PtFIT was involved in the iron deficiency reaction (Zhai et al., 2018). Ogo et al. (2006) found that overexpression of *OsIRO2*/*OsbHLH056* could promote the absorption of iron in rice. Zhao et al. (2014) demonstrated that Chrysanthemum CmbHLH1 promoted iron absorption through H^+^-ATPase mediated rhizosphere acidification. Recent research demonstrated that the overexpression of *GmbHLH57* and *GmbHLH300* up-regulated the iron absorption genes and increased the iron content of transgenic soybean plants (Li L. et al., 2018). Moreover, overexpression of *NtbHLH1* results in longer roots, altered rhizosphere pH, and increased ferric-chelate reductase activity under iron deficient conditions (Li et al., 2020). Wang et al. (2020) characterized the role of a novel rice bHLH type transcription factor OsbHLH156 in Fe homeostasis and found that *OsbHLH156* is mainly expressed in roots and transcription is greatly increased by iron deficiency. Loss of function of OsbHLH156 resulted in Fe-deficiency-induced chlorosis and reduced Fe concentration in the shoots under upland or Fe(III) supplied conditions.

Plants must maintain the homeostasis of Fe and Cu. When plants are under Fe-deficiency conditions, bHLH IVC not only directly activates *bHLH Ib* expression but also promotes *bHLH Ib* and *FIT* gene expression through interaction with bHLH121. FIT and bHLH Ib members initiate transcription of Fe-uptake genes (*IRT1* and *FRO2*) and Cu-uptake genes (*COPT2*, *FRO4*, and *FRO5*). An increase in Cu concentration alleviates Fe-deficiency stress. Under Cu-deficiency, Cu-uptake genes (*COPT2*, *FRO4*, and *FRO5*) are activated in response to SPL7. CITF1 also regulates the expression of *COPT2*, *FRO4*, and *FRO5*. SPL7 not only regulated the Cu homeostasis signaling pathway, but also suppressed (*IPON MANs*) *IMAS* and bHLH Ib of the Fe homeostasis signaling pathway (Cai et al., 2021).

### bHLHs Involved in Osmotic Stress

Proper osmoregulation is important for plant response to environmental changes (Lin et al., 2020). Drought, high salinity, low temperature, and other conditions will affect the water content of plants, thus leading to osmotic stress. Therefore, osmotic stress is often accompanied by other types of environmental stresses. Under osmotic stress, *RD29A* can be regulated through both ABA-independent and ABA-dependent pathways, thus improving plant stress resistance (Yang et al., 2016). Zhai et al. (2016) found that the expression level of *RD29A* was upregulated in transgenic plants overexpressing *TabHLH39*. The overexpression of the wild rice gene *OrbHLH2* (a homolog protein of ICE1) in *Arabidopsis* up-regulates the expression of stress response genes *DREB1A/CBF3*, *RD29A*, *COR15A*, and *KIN1*, thus enhancing tolerance to osmotic stress (Zhou et al., 2009). A MYC-type ICE1-like transcription factor *SlICE1a* in tomatoes was induced in response to osmotic stress (Feng et al., 2013).

## bHLHs Regulate Plant Flavonoids Synthesis in Response to Abiotic Stress

In vegetative tissues, the flavonoid pathway is usually induced in response to physiological and environmental fluctuations as a protective mechanism against oxidative stresses induced by pathogen infections, high-light, UV, extreme temperature, drought, salt, and deficiency of N, P, or C nutrition (Xu et al., 2015). The bHLH transcription factor family is important for regulating the biosynthetic pathway of flavonoids (Hichri et al., 2011). Flavonoids are generally divided into six categories: flavone, flavonol, isoflavone, flavanone, flavanol, and anthocyanidin (Hichri et al., 2011). In Ludwig et al. (1989) the Lc protein was initially isolated from maize and showed transcriptional activity on genes involved in anthocyanin synthesis. Heterologous overexpression of the *Lc* gene significantly enhanced the accumulation of flavonoids in mature fruits of cherry tomato (Bovy et al., 2002). Since then, the vital roles of bHLH in flavonoid synthesis have attracted growing attention. Flavonoids have strong biological activity and significant antioxidant capacity (Mitchell et al., 1998; Xu et al., 2015), and they play protective roles when plants are subjected to single or multiple stresses such as ultraviolet radiation, salt, temperature, and drought. The level of protection is determined by the position of free hydroxyl groups in the structure of flavonoids and the carbon-carbon double bond in the C ring.

To reveal the relationship between bHLH subfamilies and flavonoid biosynthesis, all the functional annotated bHLHs were categorized into the 32 known subgroups in this research (Figure 2 and Table 3). It seems that only bHLHs in subfamilies 1, 2, 5, 13, 14, 15 are involved in the regulation of flavonoid metabolism (Table 3). Among them, more than half of the annotated bHLHs involved in flavonoid metabolism regulation come from subfamilies 2 and 5. As flavonoids are important metabolites involved in plant responses to abiotic stresses, bHLHs of subfamilies 2 and 5 may regulate a series of physiological stress responses. The typical bHLH transcription factor in the IIIf subfamily [equivalent to the subfamily 5] AtTT8/AtbHLH042 can regulate the expression of *DFR* and *BAN*, two flavonoid late biosynthetic genes, thus affecting the synthesis of anthocyanins and procyanidins (Nesi et al., 2000). AtTT8/AtbHLH042 constitutes a major regulatory step in the specific activation of the expression of flavonoid structural genes (Baudry et al., 2006). Similarly, MtTT8 in *Medicago truncatula* is considered to be the central component of a ternary complex (MYB-bHLH-WD40) that controls the biosynthesis of anthocyanins and procyanidins (Li P. et al., 2016). In addition to *AtTT8*, three other bHLH genes are also categorized into the IIIf clade: AtGL3, AtEGL3, and AtMYC1. They are also known to be involved in biosynthesis of flavonoids (Maes et al., 2008; Symonds et al., 2011). Together with MYB factors, especially PAP2 (AtMYB90), GL3 seems to be a partner of bHLHs in anthocyanin accumulation under nitrogen-deficient conditions (Lea et al., 2007). In contrast, nightshade GLABRA3 (SlGL3), the homolog to AtGL3, inhibited anthocyanin accumulation in *Arabidopsis* (Tominaga-Wada et al., 2018).

**TABLE 3:** Regulation of bHLH transcription factors on metabolism of flavonoids.

Species	Nomenclature	Subfamily	Target gene	Function	References
*Zea mays* L.	Lc	5	Unknown	Synthetic anthocyanin	Ludwig et al., 1989; Bovy et al., 2002
*Arabidopsis thaliana* L.	AtTT8/AtbHLH042	5	*DFR/BAN*	Regulate the expression of *DFR* and *BAN*, Synthetic anthocyanin and procyanidine	Nesi et al., 2000; Baudry et al., 2006
*Arabidopsis thaliana* L.	AtGL3/AtbHLH001	5	E2F	Participate in the biosynthesis of flavonoids	Lea et al., 2007; Maes et al., 2008; Symonds et al., 2011
	AtEGL3/AtbHLH002	5	Unknown		
	AtMYC1/AtbHLH012	5	Unknown		
*Arabidopsis thaliana* L.	AtMYC3/AtbHLH005	2	JAZ1, JAZ3, JAZ9	Synthetic anthocyanin	Niu et al., 2011
	AtMYC4/AtbHLH004	2	JAZ1, JAZ3, JAZ9		
*Vitis vinifera* L.	VvbHLH1	1	*CBF3, RD29A*	Regulate the expression of the key enzyme genes (CHS, F3H, DFR and LDOX)	Wang et al., 2016a
*Vitis vinifera* L.	VvbHLH003	13	Unknown	Synthetic anthocyanin	Wang et al., 2018
*Vitis vinifera* L.	VvbHLH007	2	Unknown	Synthetic flavone	Wang et al., 2018
*Vitis vinifera* L.	VvMYC1	5	Unknown	Regulate the expression of the key enzyme genes (*CHI*, *UFGT*, *ANR*), synthetic anthocyanin and tannin when co-expressed with VvMYB	Hichri et al., 2010
*Medicago truncatula*	MtTT8	5	Unknown	Control anthocyanin and procyanidine biosynthesis	Li P. et al., 2016
*Solanum lycopersicum*	SlAN1	5	Unknown	Associated with anthocyanins	Li N. et al., 2018
*Solanum lycopersicum*	SlGL3	5	Unknown	Inhibit anthocyanin accumulation	Tominaga-Wada et al., 2018
*Antirrhinum majus* L.	AmDEL	5	Unknown	Induce anthocyanins	Outchkourov et al., 2014; Wang et al., 2016b
*Setaria italica*	PPLS1	5	Unknown	Regulate anthocyanin biosynthesis	Bai et al., 2020
*Malus domesica* Borkh.	MdbHLH33	15	Unknown	Control anthocyanin biosynthesis	Xu et al., 2017
*Malus domesica* Borkh.	MdMYC2	2	JAZ3	Control anthocyanin biosynthesis	An et al., 2016
*Malus domesica* Borkh.	MdbHLH74	14	Unknown	inhibit anthocyanin accumulation	Li W.F. et al., 2019
*Fragaria ananassa* Duch.	FabHLH25	2	Unknown	Synthetic anthocyanin	Zhao et al., 2018
	FabHLH29	5			
	FabHLH80	2			
	FabHLH98	5			
*Triticum aestivum* L.	TaMYC1	5	Unknown	Activate transcription of the anthocyanin biosynthesis structural genes	Shoeva, 2018
*Triticum aestivum L.*	TaPpb1	5	Unknown	Regulate anthocyanin synthesis when co-expressed with TaPpm1	Jiang et al., 2018
*Anthurium andraeanum* Linden	AabHLH1	5	Unknown	Regulate the accumulation of procyanidins when co-expressed with AaMYB3	Li L. et al., 2019
*Prunus persica*	PpbHLH3	1	Unknown	Form complexes that then regulate the synthesis of anthocyanins	Ravaglia et al., 2013
*Solanum melongena L.*	SmbHLH13	15	*SmCHS/SmF3H*	Control anthocyanin biosynthesis	Babitha et al., 2015
*Chimonanthus praecox (Linn.) Link*	CpbHLH13	2	Unknown	Reduce the anthocyanin contents	Aslam et al., 2020
*Chrysanthemum morifolium Ramat.*	CmbHLH2	5	*CmDFR*	Upregulate the CmDFR promoter and triggered anthocyanin accumulation when co-expressed with CmMYB6	Xiang et al., 2015
*Actinidia chinensis Planch*	AcbHLH42	orphan	*AcF3GT1/AcANS*	Synthetic anthocyanin when co-expressed with AcMYB123	Wang et al., 2019
*Plagiochasma appendiculatum*	PabHLH1	5	Unknown	Activate the synthesis of flavonoids and anthocyanins	Zhao et al., 2019
*Dendrobium hybrids*	DhbHLH1	5	Unknown	Regulate anthocyanin production	Li et al., 2017

Homologous or heterologous overexpression of *bHLHs* can exhibit a significant regulatory effect on the synthesis of various flavonoids. Actually, most bHLH have been found to possess a positive regulatory function on plant flavonoid synthesis. In *Arabidopsis*, transgenic plants overexpressing *AtMYC3* and *AtMYC4* showed higher levels of anthocyanin than that of wild-type plants (Niu et al., 2011). Heterologous overexpression of the grape *VvbHLH1* gene increases the activity of key enzymes in the flavonoid synthesis pathway such as Phenylalanine Ammonia-Lyase (PAL), Chalcone Synthase (CHS), Chalcone Isomerase (CHI), and Flavanone-3-Hydroxylase (F3H) as well as enhancing the salt and drought tolerance of transgenic *Arabidopsis* seedlings (2016a). VvbHLH003 and VvbHLH007 were also found to be related to anthocyanin or flavonoids synthesis (Wang et al., 2018). Likewise, the heterologous expression of eggplant (*Solanum melongena*) *SmbHLH13* can promote anthocyanin biosynthesis by positively regulating the expression of the structural genes *SmCHS* and *SmF3H*. The heterologous expression of *Arabidopsis PAP2* induces anthocyanin accumulation in tomato (Li N. et al., 2018), while the heterogeneous overexpression of liverwort (*Plagiochasma appendiculatum*) *PabHLH1* in *Arabidopsis* can activate the synthesis of both flavonoids and anthocyanins, through the up-regulation of early and late structural genes in the synthesis pathway of flavonoids (Zhao et al., 2019). The ectopic overexpression of apple *MdMYC2* also significantly up-regulated the transcriptional expression of these structural genes in transgenic Arabidopsis lines (An et al., 2016). Recent studies have shown that both triticale (*Triticum* × *Secale*) TsMYC2 and wheat (*Triticum aestivum*) *TaMYC1* can regulate anthocyanin biosynthesis and control the grain properties (Zong et al., 2019). On the other hand, several bHLHs could negatively regulate flavonoid synthesis. For example, the wintersweet (*Chimonanthus praecox* L.) *CpbHLH13* was found to reduce the anthocyanin content when ectopically overexpressed in the tobacco inflorescence (Aslam et al., 2020).

It is generally believed that the mechanism of action of flavonoids in the stress-resistance process is through their antioxidant properties. Under abiotic stresses, such as low temperature, drought, high salt, or heavy metal exposure, a series of physiological and biochemical changes will occur in plant cells, which are mainly manifested as the reduction of photosynthesis efficiency and the generation of a large number of reactive oxygen radicals, as well as serious damage to cell structure (Winkel-Shirley, 2001). The oxidative stress generated by these reactive oxygen species can also induce a large amount of flavonoids synthesis, thereby quenching the reactive oxygen species and protecting cells from oxidative damage (Treutter, 2006). Qin et al. (2021) found that abiotic stress promotes the synthesis of anthocyanins by inhibiting SPL9 through miR156, thereby facilitating the neutralization of reactive oxygen species (ROS) and simultaneously inducing *ZmSRO1e*. ZmSRO1e interacts with ZmPL1/AtPAP1 to inhibit the formation of an activated MBW complex, thus repressing the over-accumulation of anthocyanins under abiotic stress. These two pathways balance the relationship between development and abiotic stress tolerance via their control of ROS accumulation. Overexpression of the *Antirrhinum majus* bHLH gene *AmDEL* led to the up-regulation of genes related to the biosynthesis of flavonoids, proline biosynthesis, and reactive oxygen scavenging under both salt and drought stress (Wang et al., 2016b). Moreover, flavonoids can also bind with copper irons to reduce the toxic damage of these ions to cytoplasmic structures and organelles such as chloroplasts (Rice-Evans et al., 1996). The role of flavonoids in plant stress tolerance is not only limited to the removal of reactive oxygen species, but also can act as signaling molecules (Ribeiro et al., 2015).

## Concluion

As one of the most numerous TFs in eukaryotes, the bHLH family has many members and diverse functions. Many studies have shown that bHLHs can regulate plant resistances to various abiotic stresses (Babitha et al., 2015; Huang and Dai, 2015; An et al., 2016; Jin et al., 2016). As well, there are many reports that show that flavonoids in plants are synthesized in large quantities to effectively eliminate ROS, to enhance the plant’s tolerances to survive in an adverse environment. This naturally leads to an interesting question: Can bHLHs regulate plant tolerance by regulating the synthesis of flavonoids? A large number of studies have confirmed that bHLHs are involved in the synthesis of flavonoids. Specially, bHLHs in subfamily 1, 2, 13, and 15 could bind to G-box or E-box in the promoter of cold, drought, and salinity responsive genes (Tables 1, 2); members of this subfamily also could modulate the synthesis of some flavonoids (Table 3). Since members of this group share similarly conversed protein motifs (Supplementary Figure 1), it is reasonable to hypothesize that plant bHLHs in subfamily 1, 2, 13, and 15 could bind to G-box or E-box of cold, drought and salt responsive genes to further regulate the synthesis of flavonoids. Similarly, it also makes sense that bHLHs in subfamily 5 could regulate the synthesis of flavonoids to resist salinity and drought stresses (Tables 1-3). However, these hypotheses still need to be verified.

## Author Contributions

YQ, TZ, YY, JY, JX, and LG analyzed the phylogenetic relationships of bHLH family members. EP conceived the original idea for the review. All authors wrote the manuscript.

## Conflict of Interest

The authors declare that the research was conducted in the absence of any commercial or financial relationships that could be construed as a potential conflict of interest.
