# Supplementary material for: Regulatory Mechanisms of bHLH Transcription Factors in Plant Adaptive Responses to Various Abiotic Stresses
Source: Front Plant Sci. 2021 Jun 18;12:677611. doi: 10.3389/fpls.2021.677611 (PMC8250158; doi:10.3389/fpls.2021.677611)
Supplement: Supplementary file 1 [file Supplemental Figure S1.PDF › Supplemental Figure S1.PDF]

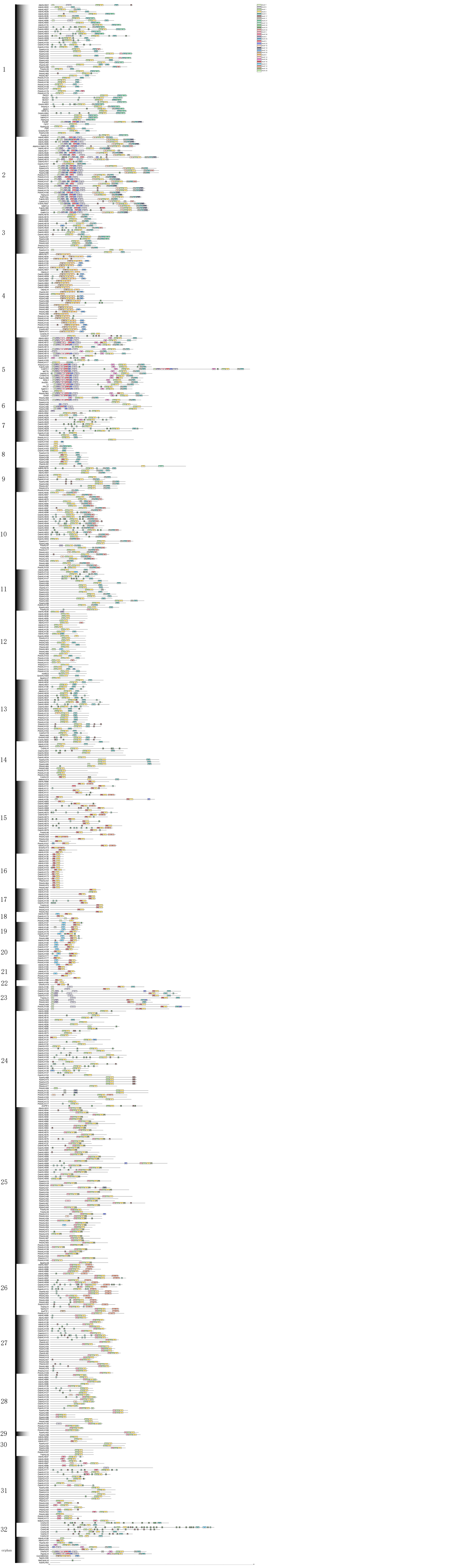
1
2
3
4
5
6
7
8
9
10
11
12
13
14
15
16
17
18
19
20
21
22
23
24
25
26
27
28
29
30
31
32
orphan
